# Day-to-day discovery of preprint–publication links

**DOI:** 10.1007/s11192-021-03900-7

**Published:** 2021-04-18

**Authors:** Guillaume Cabanac, Theodora Oikonomidi, Isabelle Boutron

**Affiliations:** 1 Computer Science Department, IRIT UMR 5505 CNRS, University of Toulouse, 118 route de Narbonne, 31062 Toulouse cedex 9, France; 2 Inserm, Université de Paris, Centre of Research in Epidemiology and Statistics (CRESS), 75004 Paris, France; 3 Centre d’épidémiologie Clinique, AP-HP, Hôpital Hôtel Dieu, 75004 Paris, France; 4 Cochrane France, 75004 Paris, France

**Keywords:** Data linking, Preprint, Publication, Living systematic review, COVID-19

## Abstract

Preprints promote the open and fast communication of non-peer reviewed work. Once a preprint is published in a peer-reviewed venue, the preprint server updates its web page: a prominent hyperlink leading to the newly published work is added. Linking preprints to publications is of utmost importance as it provides readers with the latest version of a now certified work. Yet leading preprint servers fail to identify all existing preprint–publication links. This limitation calls for a more thorough approach to this critical information retrieval task: overlooking published evidence translates into partial and even inaccurate systematic reviews on health-related issues, for instance. We designed an algorithm leveraging the Crossref public and free source of bibliographic metadata to comb the literature for preprint–publication links. We tested it on a reference preprint set identified and curated for a living systematic review on interventions for preventing and treating COVID-19 performed by international collaboration: the COVID-NMA initiative (covid-nma.com). The reference set comprised 343 preprints, 121 of which appeared as a publication in a peer-reviewed journal. While the preprint servers identified 39.7% of the preprint–publication links, our linker identified 90.9% of the expected links with no clues taken from the preprint servers. The accuracy of the proposed linker is 91.5% on this reference set, with 90.9% sensitivity and 91.9% specificity. This is a 16.26% increase in accuracy compared to that of preprint servers. We release this software as supplementary material to foster its integration into preprint servers’ workflows and enhance a daily preprint–publication chase that is useful to all readers, including systematic reviewers. This preprint–publication linker currently provides day-to-day updates to the biomedical experts of the COVID-NMA initiative.

## Introduction

The World Health Organization declared the coronavirus disease 2019 a ‘public health emergency of international concern’ on January 30, 2020. COVID-19 was soon qualified as a pandemic in March.^1^ Worldwide researchers in biomedicine and many other fields instantly turned their attention and devoted much efforts to this critical issue. Because peer review in biomedical journals usually takes more than 5 months from submission to publication (Abdill and Blekhman 2019, p. 9), scientists *en masse* resorted to preprint servers for quicker result dissemination. Authors posting—and then updating—preprints fuelled the fast communication of ongoing experiments and preliminary results that could help guide policy and clinical decision-making.

Fraser et al. (2020a) stressed the unprecedented role of preprints in the dissemination of COVID-19 science. In four months, thousands of preprints were posted to *medRxiv* (Rawlinson and Bloom 2019) and *bioRxiv* (Sever et al. 2019) mainly. Results also appeared in 29 other preprint servers but to a lesser extent. Meanwhile, journals prioritized COVID-19 submissions and organised faster peer review with a median time of 6 days from submission to acceptance (Palayew et al. 2020). By the end of April, there were 16,000 publications on COVID-19, more than 6000 of which were manuscripts hosted on preprint servers (Fraser et al. 2020a).^2^ As a result, some research results have appeared in both preprint articles (sometimes under multiple successive versions) and peer-reviewed articles. The evidence conveyed by each version is to be understood with an evolving context: studies involving more patients as time passes produce conclusions changing over time, for instance. Such changes in conclusions between a preprint and its published counterpart were underlined in recent studies (Fraser et al. 2020a; Oikonomidi et al. 2020).

Linking the various versions of a research work—from preprint to published in a peer-reviewed venue—is crucial for readers looking after the latest and most trustworthy evidence. This preprint–publication linking issue is as old as preprint servers themselves, *arXiv* being one of the oldest as introduced in 1991 (Ginsparg 1994; McKiernan 2000). This has become a key issue with the COVID-19 preprint avalanche: scientists have little clue about the final status of a preprint: has it been published in a peer-reviewed venue yet? Our paper tackles this open issue to provide readers with the complete lineage of a research work. We comb the literature to weave links from drafts posted on whatever preprint server to any subsequent publication in whatever peer-reviewed venue, such as journals, books, and conferences proceedings.

## Problem statement: why we need a day-to-day preprint–publication linker?

Most preprint authors submit their work to peer-reviewed journals (Abdill and Blekhman 2019). The peer-reviewers’ comments and critiques lead authors to revise their manuscripts, substantially at times. These changes get incorporated in the subsequently published journal article and the preprint is not the latest version of the work any more. Readers should refer to the journal publication for the latest peer-review certified results instead.

The COVID-19 preprint avalanche challenged the preprint servers that enforce expert-based screening procedures (Kwon 2020). Several curation initiatives were launched to tame the incessant flow of literature doubling every 20 days as of May (Brainard 2020). We are contributing to one of these, called COVID-NMA^3^: the Cochrane living systematic review and network meta-analysis on COVID-19 (Boutron et al. 2020a, b, c). A living systematic review provides a frequently updated report on a specific research question (Elliott et al. 2014; Ravaud et al. 2020). The COVID-NMA consortium performs daily bibliographic database searches to identify relevant newly-published literature assessing preventive, therapeutic, and post-acute care interventions for COVID-19. The data extracted from newly-identified publications are then rapidly incorporated in the evidence synthesis, which is updated once a week. Monitoring preprint versions and tracking all subsequent publications in peer-reviewed venues proves crucial to reflect knowledge updates to the living systematic review.

The next section reviews how preprint servers and bibliometric studies sought to link preprints to publication. We stress their limitations, which motivates the introduction of a new preprint–publication linking algorithm.

## Related work on preprint–publication linking

Several stakeholders have been striving to link preprints to subsequent publications. We discuss the attempts of 1) Crossref as the leading DOI registration agency for scholarly documents, 2) the organisation running *bioRxiv* and *medRxiv*, and 3) researchers publishing bibliometric studies. We stress the shortcomings of these attempts regarding a day-to-day preprint–publication linking task, which motivates our approach.

### Publication–preprint linking at Crossref

Crossref is one of the ten DOI registration agencies.^4^ It has minted 106 million DOIs for 13 types of documents, with journal publications and scholarly book representing the largest part of these (Hendricks et al. 2020). Himmelstein et al. (2018, p. 15) estimated that ‘the overwhelming majority of DOI-referenced scholarly articles are registered with Crossref.’ They started minting DOIs for preprints in 2016 (Lammey 2016).

**Fig. 1:**
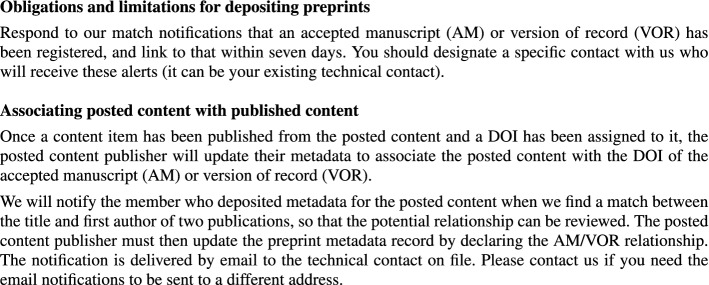
Crossref documentation on preprint metadata updates expected from content publishers. Excerpt of the *Introduction to posted content (including preprints)* available from https://www.crossref.org/education/content-registration/content-types-intro/posted-content-includes-preprints/

Crossref monitors the published literature to link preprints to publications based on matching titles and first authors (Fig. 1). They send potential preprint–publication matches to DOI registrants (e.g., the *medRxiv* maintainers) who are requested to diligently show the publication DOIs along with their preprints: ‘all preprints need to link to a resulting journal article when they are alerted by Crossref that one exists’ (Hendricks et al. 2020, p. 418). However, a Crossref audit of preprint metadata acknowledged ‘incomplete member data’ as some publishers failed to ‘provide links to published articles in their metadata’ (Lin and Ram 2018). Some preprint servers, though, strive to identify their preprints that were subsequently published in a peer-reviewed venue, as discussed in the next section.

### Publication–preprint linking at *bioRxiv* and *medRxiv*

**Fig. 2:**
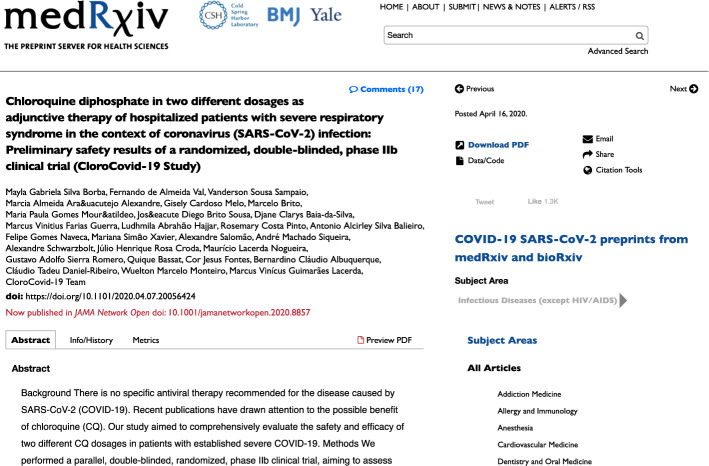
The *medRxiv* preprint doi:10.1101/2020.04.07.20056424 with linked paper in *JAMA Network Open*

The Cold Spring Harbor Laboratory^5^ launched and runs the two flagship preprint servers in biomedicine: *bioRxiv* and *medRxiv* (Rawlinson and Bloom 2019; Sever et al. 2019). Each deposited preprint is associated to a DOI minted by Crossref. Preprints may be updated, with all intermediate versions kept: v1 is the initial submission and updates are sequentially named v2, v3, and so on. The preprint DOI always resolves to the latest deposited version. Preprint pages prominently link to any subsequent journal publication (see the red DOI link in Fig. 2). Staff at *medRxiv* infer these DOIs and ask preprint authors for confirmation, as explained in the FAQ (medRxiv 2020):

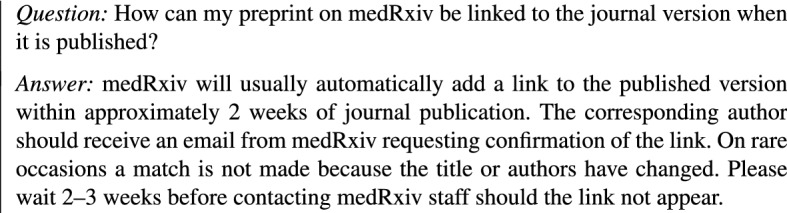



This process is not further documented for *medRxiv* albeit (Sever et al. 2019, p. 4) indicate using ‘a variety of scripts that search PubMed and Crossref databases for title and author matches’ for *bioRxiv*. The literature stressed a limited coverage of the actual preprint–publication links. A study found 37.5% of missing publication links for 120 *bioRxiv* preprints incorrectly reported not to be published (Abdill and Blekhman 2019, p. 6–8). The same test on 12,788 *bioRxiv* preprints yielded 7.6% of missing publication links (Fraser et al. 2020b, p. 621).

Working at the COVID-NMA living systematic review (Boutron et al. 2020a, b, c), we also noticed many preprint–publication links that failed to be reported on *medRxiv*. Among the 323 *medRxiv* preprints we were monitoring as of 23 October 2020, we managed to find a publication for 116 of these whereas *medRxiv* provided 48 links to publications only (41.4%). This means that readers are unaware of the latest peer-reviewed results for more than half of the preprints related to COVID-19.

### Publication–preprint linking in various bibliometric studies

Recent bibliometric studies questioned the outcomes of preprints as peer-reviewed publications. They collected preprint–publication links from the online records of preprint servers reporting publication DOIs when available. Such studies mined *bioRxiv* (Abdill and Blekhman 2019; Abdill et al. 2020; Anderson 2020; Fraser et al. 2020b), *RePEc* in the socio-economic sciences (Baumann and Wohlrabe 2020), and one of the oldest preprint servers: the *arXiv* (Klein et al. 2019; Lin et al. 2020; Gao et al. 2020). Aforementioned caveats were raised as preprint servers failed to signal publication DOIs exhaustively (Abdill and Blekhman 2019; Fraser et al. 2020b).

Other studies searched for ‘published preprints’ in the Web of Science (WoS) and Scopus, the two leading subscription-based bibliographic sources. Larivière et al. (2014) sought the title and first author of *arXiv* preprints in the 28-million records of an in-house copy of the WoS. They used fuzzy string matching to accommodate for minor differences in the character strings being compared. Fraser et al. (2020b) used a similar strategy on an in-house copy of Scopus. Eventually, some studies relied on Crossref. For instance, Lin et al. (2020) matched preprints to an in-house copy of 40-million Crossref records after training a Bidirectional Encoder Representations for Transformers (BERT) model.

In brief, there are two methods to collect preprint–publication links. On the one hand, preprint servers report such links but not exhaustively, at least for *bioRxiv*. On the other hand, one may mine bibliographic sources (e.g., WoS, Scopus) with preprint features as query, including titles and authors. This approach has the three following drawbacks, though. First, the providers of these sources update them regularly but a delay remains between the publication (in early view or in print) and the inclusion into bibliographic indices. This is problematic for any day-to-day screening of the literature. Second, each update of a bibliographic source must be accounted for to perform preprint–publication linking on fresh data. This requires the downloading of huge bibliographic datasets and their subsequent indexing, a computationally intensive and time-consuming task. Third, the WoS and Scopus are known to index a selected fraction of the published literature only (Visser et al. 2020).

The strategy we designed alleviates these limitations. It does not require any prior downloading and indexing of any bibliographic data. It does not require any further data update either. Relying on search queries submitted to the Crossref API, it operates on one of the most comprehensive and fresh index of the peer-reviewed literature.

## Contribution: designing and benchmarking the preprint–publication linker

This section introduces the algorithm we designed for the day-to-day discovery of preprint–publication links. We first consider the links already established by *medRxiv* and gather knowledge about the most successful features to match publications to preprints. These features inform our original ‘search and prune’ strategy leveraging the Crossref API as a third-party academic search engine. The source code of the linker is released as supplementary material (Appendix 1) so that readers can replicate our results or seek new preprint–publication links in *medRxiv* or in any other preprint servers.

### Collecting the *medRxiv*-established preprint–publication links

**Fig. 3:**
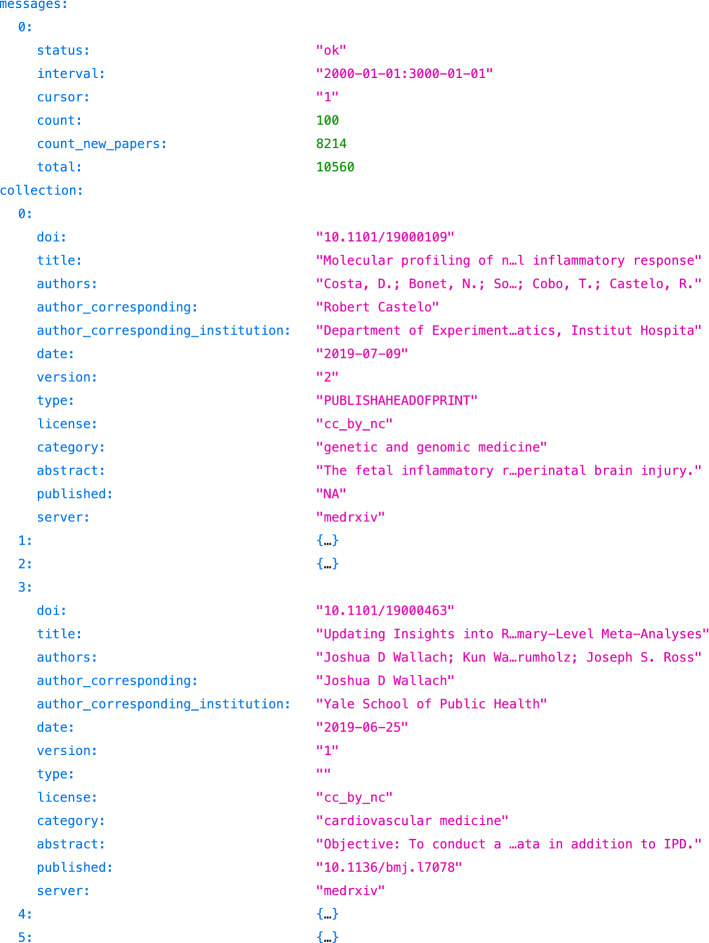
Excerpt of the listing of all *medRxiv* preprints, available from https://api.biorxiv.org/details/medrxiv/2000-01-01/3000-01-01/1 in JSON format. Each preprint comes with its associated metadata (e.g., title, authors, version) and is optionally linked to a publication (e.g., see the third record with 10.1136/bmj.l7078)

The Cold Spring Harbor Laboratory operating both *bioRxiv* and *medRxiv* offers an Application Programming Interface (API)^6^ for programmatic access to the data hosted in both servers. We used it to collect the preprint–publication pairs for all *medRxiv* preprints. Figure 3 shows an excerpt of the resulting records: one per preprint version. As of July 14, *medRxiv* hosted 10,560 preprint versions corresponding to 8214 unique preprints. Filtering these records on the published field, we found 741 preprints with one linked publication.

We then retrieved preprint and publication metadata by querying the Crossref API^7^ with the DOIs listed earlier. Crossref provides the bylines with the ORCID of each author when available and each author’s complete identity. First names are given in full, which is more precise than initials for some *bioRxiv* preprints (see Costa, D. in Fig. 3). One DOI failed to resolve (10.34171/mjiri.34.62) and we excluded the associated pair from the collection, which thus comprises 740 preprint–publication links.

### Designing features to match publications with preprints

Based on the retrieved metadata for the 740 preprint–publication pairs, we designed three features to be used as criteria to match a candidate publication to a given preprint. The next sections detail the rationale and implementation of these features based on the timeline, title, and byline matching.

#### Timeline matching

According to the FAQ (medRxiv 2020), the first version of a preprint should predate the acceptation date of the linked publication:

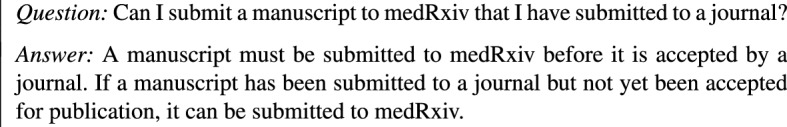



Among the 740 collected *medRxiv* preprints, less than one percent ($N=5$) do not comply with this requirement (Table 1). This observation suggests that searching for publications with an acceptance date posterior to the preprint’s submission date works in most cases.

**Table 1:** Five outlying *medRxiv* preprints posted after the acceptation date of the linked publication

medRxiv preprint		Publication	
DOI	Version 1	DOI	Acceptation
10.1101/19009456	18 Nov. 2019	10.1093/annonc/mdz261.007	1 Oct. 2019
10.1101/19013318	29 Nov. 2019	10.1016/j.ahj.2019.11.011	22 Nov. 2019
10.1101/2020.02.27.20028647	29 Feb. 2020	10.1093/annonc/mdz252.055	1 Oct. 2019
10.1101/2020.04.30.20086736	5 May 2019	10.1172/jci.insight.138999	23 Apr. 2020
10.1101/2020.05.09.20082909	15 May 2020	10.7554/elife.58728	11 May 2020

#### Title matching

We hypothesised that the title of a preprint (in its latest version) and the title of its associated publication are likely to be very similar. Running through the 740 paired titles, we noticed that minor variations often occur. Some typographic markers differ between preprint and publication versions: hyphens get typeset as em- or en-dashes, for instance. In addition, acronyms in preprint titles are sometimes expanded in the publication counterparts. For instance, the strings *USA* and *US* were likely to appear as *the United States of America* and a few occurrences of *SARS-CoV-2* were changed to *severe acute respiratory syndrome coronavirus 2*.

We used a 3-step method to measure the similarity between a preprint’s title and its associated publication’s title. First, both titles were pre-processed to expand acronyms and uniformise typographic markers. Second, the resulting titles were tokenised using whitespace as delimiter. Third, the Jaccard distance between the two resulting token lists was computed (Levandowsky and Winter 1971) to reflect the share of words in common compared to all words occurring in the preprint and publication titles. The resulting similarity value is the one-complement of this distance.

Perfect similarity occurred for 81% ($N=600$) of the 740 preprint–publication pairs. A similarity of 80% or more characterises 90% ($N=626$) of the pairs. A small fraction of 8% ($N=58$) of the pairs show a [0.5, 0.8[ similarity. One pair only has a similarity below 10%: the preprint title was recast before submission to the *British Medical Journal*. This example of a 5% inter-title similarity features very little words in common:

- The preprint 10.1101/2020.05.02.20086231 in its latest version was titled: *Trends in excess cancer and cardiovascular deaths in Scotland during the COVID-19 pandemic 30 December 2019 to 20 April 2020*. (We note in passing that the metadata differs slightly from the title given in the PDF version of the preprint).
- The subsequent publication 10.1136/bmj.m2377 was titled: *Distinguishing between direct and indirect consequences of covid-19*.

These tests suggest that most preprint–publication pairs show high to perfect similarity. Setting a 10% lower bound for inter-title similarities should filter irrelevant pairs out.

#### Byline matching

We hypothesised that the first author of a preprint (in its latest version) remains as first author in the published paper. There is only one counterexample among the 740 pairs: the first author of preprint 10.1101/2020.03.03.20030593 becomes third author in the associated publication 10.1001/jama.2020.6130 promoting the preprint authors ranked 10 and 2.

Comparing the ORCIDs of the preprint vs. publication first author is the most effective way when ORCIDs are provided. This occurred for 30% ($N=219$) of all pairs. As a fallback solution, we compared the identity (i.e., last name and first name) of paired authors. We noted several discrepancies hindering any matching on strict string equality, such as:

- Typographic variations w.r.t. accentuated letters and dashes: author ‘Ana Fernandez-Cruz’ of preprint 10.1101/2020.05.22.20110544 appears as ‘Ana Fernández Cruz’ in publication 10.1128/aac.01168-20.
- Corrected last name: author ‘Goldstein, E.’ of preprint 10.1101/19012856 appears as ‘Goldsteyn, E.’ in publication 10.17513/mjpfi.12945.
- Corrected first name: author ‘Achakzai, Mohammad’ of preprint 10.1101/19001222 was changed to ‘Achakzai, Muhammad I.’ in publication 10.3390/jcm8122080. Note that besides the changed letter, a middle initial was added.
- Collective name: first author ‘Korea Centers for Disease Control and Prevention COVID-19 National Emergency Response Center’ of preprint 10.1101/2020.03.15.20036350 is reworded as ‘COVID-19 National Emergency Response Center, Epidemiology and Case Management Team, Korea Centers for Disease Control and Prevention’ in publication 10.24171/j.phrp.2020.11.2.04.

We designed an author–matcher algorithm that compares two authors’ ORCIDs or, when not available, their identity. Hyphens and accents were removed to uniformise the strings. Then, the family names and up to the top three letters of the first names were compared, as a way to overcome changes in middle initials. Tested on the 740 pairs, this approach showed a 97% ($N=721$) success rate. This suggests that first author comparison is effective for preprint–publication matching.

We tested another criterion that proved less effective: the number of preprint vs. publication authors. It appeared that 95% ($N=708$) pairs validate the following hypothesis: the number of publication authors is equal or greater than the number of preprint authors. We disregarded this criterion when combining the other more effective ones as presented in the next section.

### Feature benchmarking on the *medRxiv* gold collection of ‘published preprints’

We combined these features to form a burden of proof, which is used to decide when a preprint–publication pair should be reported. The $match (p,j)\in {\mathbb {B}}$ boolean function is true when a journal paper *j* is likely to be linked to a preprint *p*, such as:

1 $$\begin{aligned} \begin{aligned} match (p,j)&= simTitles (p,j)\geqslant 0.8\\&\,\vee \Big ( simTitles (p,j)\geqslant 0.1\\&\quad \,\wedge matchDates (p,j)\\&\quad\, \wedge \big ( matchORCIDs (p,j) \vee matchFirstAuthors (p,j)\big )\Big ) \end{aligned} \end{aligned}$$

where:

- $simTitles (p,j)\in [0,1]$ is the one-complement of the Jaccard distance between the titles.
- $matchDates (p,j)\in {\mathbb {B}}$ is true when the date of *p* is earlier of equal to the date of *j*.
- $matchORCIDs (p,j)\in {\mathbb {B}}$ is true when the ORCIDs of the first authors are identical.
- $matchFirstAuthors (p,j)\in {\mathbb {B}}$ is true when the identifiers of the first authors match.

Titles showing a 80% or higher similarity were found to be excellent evidence. This criteria circumvents the aforementioned timeline issues for the five problematic cases of Table 1 and for 18 out of 19 preprint–publication cases with non-matching first authors.

For titles with less than 80% similarity, candidate pairs should have title similarity of 10% at least, compatible dates (i.e, a preprint should be posted before its journal counterpart acceptance), and identical first authors (based on either ORCIDs or identity comparisons).

We applied equ. 1 on the 740 preprint–publication pairs of the *medRxiv* gold collection. The matching is almost perfect with 99% validated pairs ($N=738$). Failure analysis on the two missed pairs showed that:

- 10.1101/2020.03.03.20030593 and 10.1001/jama.2020.6130 have little title similarity (31%) and the preprint first author is third author in the subsequent publication.
- 10.1101/2020.05.02.20086231 and 10.1136/bmj.m2377 have poor title similarity (5%) and the middle initial of the first author is only present on the publication, which impaired identity matching.

The next section discusses the implementation of the tested search features as input parameters to the Crossref API and post-processing filters.

### Implementation of the search features using the Crossref API

As a reminder, we tackle the following information retrieval task: for a given preprint, find all subsequently published articles. We need to comb the most comprehensive and up-to-date scholarly literature, looking for publications matching the features of the preprint under consideration. This section describes the preprint–publication linker we designed. It combs the scholarly literature for publications matching preprints using the daily-updated Crossref bibliographic source that comprised 117 million records as of October 2020.^8^

We designed a two-step ‘query and prune’ process to retrieve any publication likely to be a follow-up of a given preprint.

First, the program queries the Crossref REST API with the parameters in Table 2. These reflect the features that we established and tested against the *medRxiv* gold collection of ‘published’ preprints. Exclusion filters delineate the search space based on two criteria. First, the publication’s date must be posterior or equal to the preprint’s first version. Second, the publication’s type must include materials published in journals, proceedings, and books. Crossref’s search engine uses a ‘best match’ approach to retrieve up to 20 records based on title and byline similarity. Each returned record comes with a score reflecting the similarity between the query (i.e., preprint) and the matching publication.

**Table 2:** Searching the literature for publications matching a given preprint: invocation of the Crossref REST API at https://api.crossref.org with parametrised works resource (see https://github.com/CrossRef/rest-api-doc#parameters)

Parameter	Value
filter	(from-created-date: Earliest date (v1) of preprint submission)
	and
	(type is journal-article or proceedings-article
	or book-chapter or book-part or book-section)
query.bibliographic	The title of the preprint (latest version available)
query.author	The authors of the preprint (latest version available)
sort	score
order	desc
rows	20
select	author,container-title,created,DOI,score,title

**Fig. 4:**
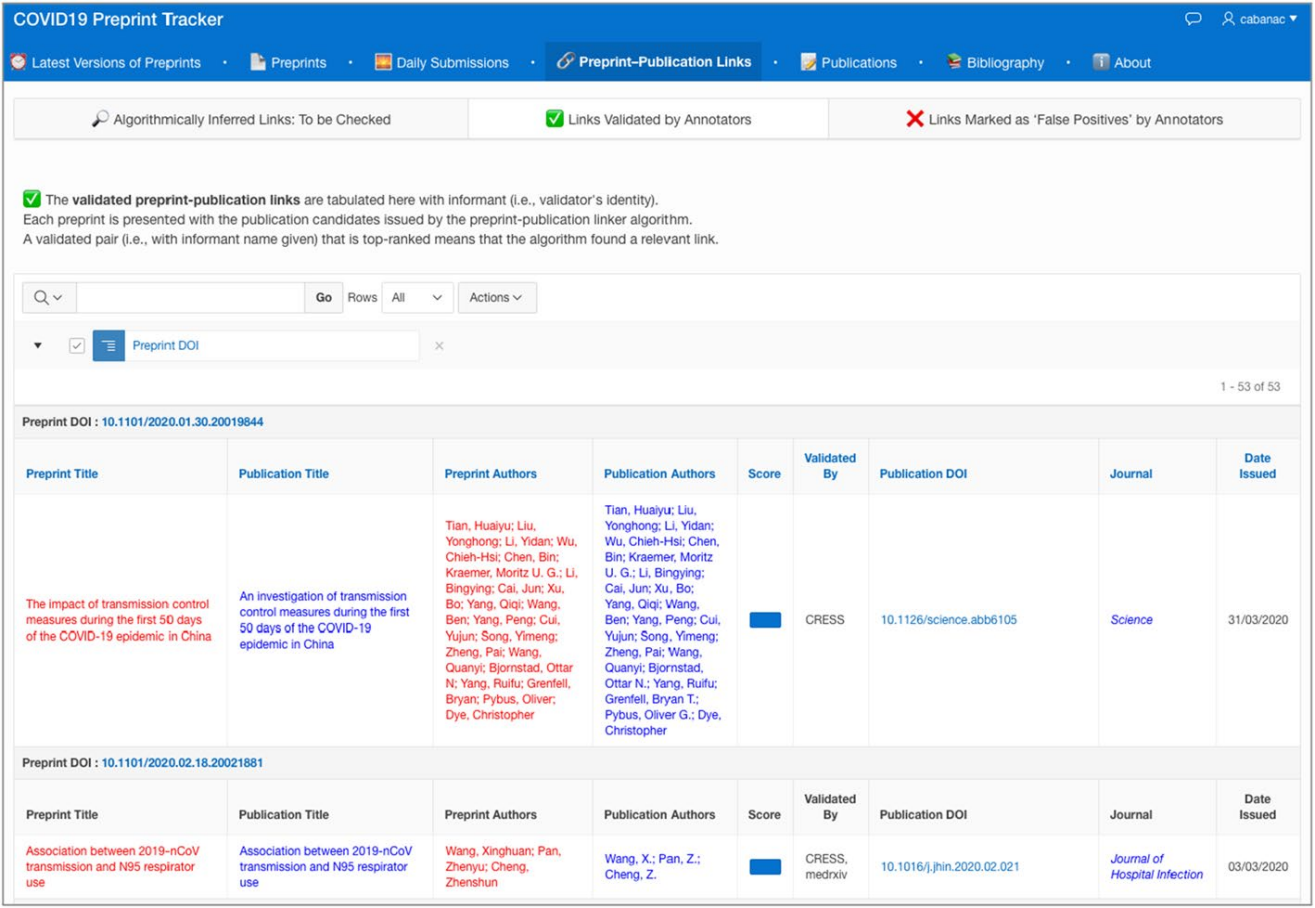
Validated preprint–publication links shown on the ‘COVID19 Preprint Tracker’ used by COVID-NMA and hosted at https://www.irit.fr/~Guillaume.Cabanac/covid19-preprint-tracker

Second, the program prunes the publication records that are unlikely to be preprint follow-ups. Equation 1 is applied to discard publications whose titles and bylines fail to match those of the preprint under consideration. A final filter rejects Elsevier records from the *Social Science Research Network* (SSRN) preprint server whose DOIs starting with 10.2139/ssrn were incorrectly deposited with the journal-article type despite being preprints (Lin and Ram 2018). The surviving record(s) are shown to the user who is expected to validate the preprint–publication pair(s) tabulated by decreasing matching likelihood (Fig. 4).

## Evaluation of the preprint–publication linker

This section introduces the test collections used to benchmark the preprint servers and the proposed preprint–publication linker.

### Test collection of 343 preprints on COVID-19

We built a test collection with the 343 preprints curated for the COVID-NMA living systematic review (Boutron et al. 2020a, b, c) as of 23 October 2020 (Appendix 2). They were initially posted (version 1) in 2020 between February 2 and October 5, with May 29 as the median date.

**Table 3:** Provenance and publication status of the 343 preprints in the test collection as of 23 October 2020

Preprint server	Hyperlink	Preprints	‘Published’ preprints	
			Total	Reported
*medRxiv*	https://medrxiv.org	323	116	48
*SSRN*	https://ssrn.com	13	4	0
*Research Square*	https://researchsquare.com	5	1	0
*Preprints*	https://preprints.org	2	0	0
Total		343	121	48

The number of preprints posted on each preprint server is tabulated, some of which appeared as peer-reviewed publications (total). A link to such subsequent publications was found on the preprint page for some of these ‘published’ preprints (reported)

Between February and August 2020, two epidemiologists of the COVID-NMA team independently checked preprint pages (see Fig. 2) and systematically searched and screened PubMed as well as secondary sources such as the Living Overview of Evidence (L.OVE) database by Epistemonikos^9^ and the Cochrane COVID-19 Study Register^10^ to identify preprint updates and published articles of the preprints identified for COVID-NMA (Boutron et al. 2020a, b, c). The researchers used the same search terms and study inclusion and exclusion criteria in searching for preprints and related published articles. They also used earlier versions of the preprint–publication linker to spot matches. The included preprints and published articles were then compared, using title keywords and first author names, to identify matches. In addition, they asked the corresponding authors of 272 unpublished studies for any subsequent publication as of 25 August 2020 and none of the 123 respondents reported any such publication (Oikonomidi et al. 2020). Eventually, on 23 October 2020, the second author (TO) also used a 2-step search strategy to identify publications associated to preprints deemed unpublished:

1. TO queried Google Scholar by entering the full name of the first author in the field “Return articles authored by”, combined with the name of the intervention (using the same term as reported in the preprint title, e.g., ‘lockdown,’ ‘antivirals,’ ‘remdesivir’) in the field “with all of the words” and selecting the option “in the title of the article”,dated from 2020. TO screened the search results by comparing titles and, if needed, abstracts with the preprint, to identify associated articles. TO checked all results, including articles in which the first author of the preprint had a different authorship position.
2. TO repeated this search in the L.OVE database for all preprints for which no article had been identified in the previous step. TO restricted the dataset by using the following filters: COVID-19 studies, Prevention or Treatment studies, Primary studies reporting data. TO downloaded this dataset in Excel format. For each preprint, TO used the filter function to search for the name of the first author in the authors column (in any position). Within this subset, TO then searched for the name of the intervention assessed in the study in the title column. When a potential match was identified, TO compared the titles and, if needed, abstracts to verify the preprint–article pair.

Most of the 343 preprints were posted to *medRxiv* (94.2%) and some appeared on other preprint servers: *SSRN*, *Research Square*, and *Preprints* (Table 3). A subset of 121 preprints (35.3%) were published in a peer-reviewed venue—journals only. We call these ‘published’ preprints in Table 3. Note that 3 publications appeared in journals that do not assign DOIs and 2 publications have a DOI that failed to resolve via https://doi.org. The error message ‘DOI Not Found’ suggests that the publishers failed to register these publications properly.^11^ We reported this issue via the appropriate form at doi.org so that the publishers fix it.

### Sensitivity/sensibility analysis of the preprint servers

**Fig. 5:**
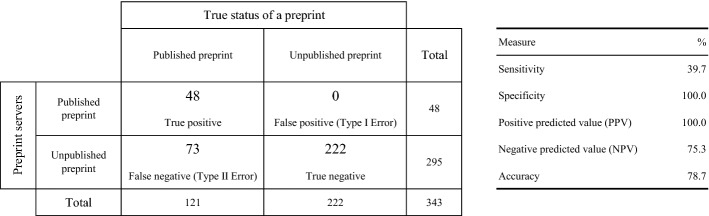
Evaluation of the preprint servers on the 343 preprint reference set, as of 23 October 2020

Overall, the preprint servers reported 39.7% of all existing publication links only (Fig. 5). This stresses the current limitation of preprint servers failing to report most of the preprint–publication links. Not finding the publication linked to a ‘published’ preprint translates into a loss in accuracy for systematic reviews which should report the latest evidence available in any peer-reviewed venue instead.

### Sensitivity/sensibility analysis of the preprint–publication linker

We ran the preprint–publication linker on the test collection on 23 October 2020. It processed the 343 preprints in 140 min, that is 2.85 min per preprint on average. The linker found matching publications for 128 preprints. Most preprints were matched to one publication only ($N=110$, that is 85.9%) whereas two to three matches were found for 18 preprints (14.1%). We kept the top-ranked publication only, results being sorted by decreasing score. A preprint–publication pair was labelled as True Positive if the publication DOI found matched the DOI that was identified by the annotators and registered in the test collection; it was labelled as False Positive otherwise.

**Fig. 6:**
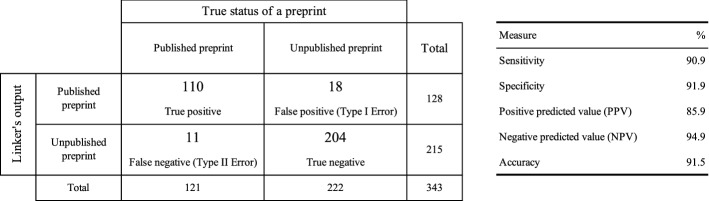
Evaluation of the preprint–publication linker on the 343 preprint reference set, as of 23 October 2020

Compared to the 78.7% accuracy of the preprint servers (Fig. 5), our linker’s accuracy of 91.5% reflects how effective it was at discovering publications related to preprints (Fig. 6). It retrieved 46 of the 48 preprint–publication pairs that preprint servers report online while managing to identify 64 additional preprint–publication links. It correctly identified ‘published’ preprints (90.9% sensitivity) and ‘unpublished’ preprints (91.9% specificity). With a 94.9% negative predictive value, most preprints that the linker marked as ‘unpublished’ truly were.

We performed a failure analysis. The 18 false positives were publications from the same research group as the preprint, working on COVID-related cases, but not directly connected to the given preprint. For one preprint–publication pair only, the relevant publication ranked second (10.1101/2020.04.27.20073379). It is interesting to note, however, that the top-ranked publication was also of interest: it is an erratum of the expected publication.^12^

In addition, the analysis of the 11 false negatives shows that the preprint–publication linker failed to retrieve:

- 3 publications in journals that do not assign DOIs.
- 2 publications with a defunct DOI that failed to resolve via https://doi.org.
- 2 publications whose first author differs from the preprint’s first author. We had identified and discussed some of these cases in the “Byline matching” section.
- 2 publications published under a Consortium name (The RECOVERY Collaborative Group) in the *New England Journal of Medicine* whereas the list of authors was given in the preprints.
- 1 publication whose title differs much from the associated preprint.
- 1 publication with an erratum, this latter being retrieved by the linker instead of the initial publication.

The 91.5% accuracy of the preprint–publication linker suggests an improvable linking process. For a recall-oriented complementary screening, after an initial screening step, users may tune parameters of the preprint–publication linker (Eq. 1) to retrieve a larger number of candidate publications, at the expense of a higher false positive rate. The fringe cases involving changes of first authors between a preprint and a publication, as well as poor inter-title overlap could be tackled that way. Another case concerns the infrequent publications with non Crossref-minted DOIs or no DOI at all that our search strategy based on Crossref fails to identify. Other bibliographic sources offering a programmatic access, such as PubMed (Schuler et al. 1996) and Dimensions (Herzog et al. 2020), could be queried for each preprint under study and results scrutinised for extra candidate preprint–publications links to assess.

### Performance of the preprint–publication linker on a larger and more diverse collection

We performed a final evaluation of the preprint–publication linker on a larger and more diverse test collection (Appendix 3). Crossref stores preprint–publication pairs for preprint servers running the whole gamut of subject areas. We sampled this set of DOI–DOI pairs to build the test collection. For each month of years 2017–2020, we queried the Crossref API for 100 randomly selected preprints issued on that month, provided each of them was associated to a publication via the is-preprint-of relation type. Most of the 4800 preprints were published by the Cold Spring Harbor Laboratory (41.2%), Research Square (16.1%), Copernicus GmbH (13.6%), the Center for Open Science (10.1%), and twelve other entities. The DOIs of linked publications were mainly minted by Crossref ($N=4693$; 97.8%) followed by DataCite ($N=96$, 2.0%) and two other registration agencies. Six DOIs were not properly registered (‘DOI Not Found’ error discussed earlier) and two records provided a URL instead of a DOI.

We fed the preprint–publication linker with the 4693 preprint DOIs whose corresponding publications had a Crossref-minted DOI. Our algorithm retrieved 1 to 16 results (median: 1) for each preprint DOI. When considering the top-ranked result only, the algorithm had a 89.62% precision. Considering the top 3 results leads to a 91.20% precision while considering the entire results leads to a 91.26% precision. The preprint–publication linker performed similarly on the COVID-NMA collection (previous section) and on a larger and more diverse test collection stemming from various preprint servers supporting several scientific communities.

## Conclusion

Signaling the preprints that eventually appeared in peer-reviewed journals proves difficult for preprint servers. (Abdill and Blekhman 2019, p. 6–8) reported 37.5% of missing publication links for 120 bioRxiv preprints incorrectly reported as unpublished. The same test on 12,788 *bioRxiv* preprints yielded 7.6% of missing publication links (Fraser et al. 2020b, p. 621). We faced the same issue when conducting a living systematic review on COVID-19: 60.3% of the ‘published’ preprints posted at *medRxiv* and 3 other servers were not presented with their associated publication. With preprinting gaining momentum (Kwon 2020), the prompt linking of publications to preprints is getting increasingly harder for preprint servers.

The preprint–publication linker we designed matches preprints with subsequently published articles. It harnesses the Crossref as an up-to-date and comprehensive source of bibliographic metadata available for free (Hendricks et al. 2020). We evaluated it on a 343 reference preprint set manually identified and curated by the COVID-NMA biomedical experts (Boutron et al. 2020a, b, c). Considering preprint servers as a baseline characterised by a 78.7% accuracy, the proposed linker yielded a 91.5% accuracy which is a 16.26% increase in accuracy for the preprint–publication linking task.

The software of the linker is released as supplementary material to help the maintainers of preprint servers who strive to find and show the publications associated to the preprints they host. Displaying a publication link on a preprint’s page contributes to inform readers on the status of a given research: from non peer-reviewed preprints to peer-reviewed publications. A more comprehensive reporting of preprint–publication links has implications for bibliometric studies, too. Some acknowledged underestimating the number of preprints that passed peer-review and were published as a journal paper (e.g., Abdill et al. 2020; Abdill and Blekhman 2019; Fraser et al. 2020a; Fraser et al. 2020b) but others failed to discuss this caveat (e.g., Anderson 2020; Homolak et al. 2020). On another note, citation count consolidation between preprints and associated publications (Gao et al. 2020) depends on a comprehensive identification of all preprint–publication links. A more accurate picture of preprint–publication links is needed to reassess the increasing role of preprints in contemporary science communication.

## Appendix 1: Supplementary materials

The code developed to collect and analyse the data reported in this article is archived at Zenodo (10.5281/zenodo.4432116) and available at https://github.com/gcabanac/preprint-publication-linker.

## Appendix 1: Collection of *medRxiv* preprint–publication links and feature analysis

The medRxiv preprint–publication links were collected with the medrxiv-gold-collector.py script. It was run on 14 July 2020 and produced medrxiv-gold.tsv. The medrxiv-gold-analyzer.py script compared preprint and publication features, producing medrxiv-gold-analyzer.tsv that was further analysed in the 20200916-medRxiv_analysis.xlsx spreadsheet.

## Appendix 2: Evaluation of the preprint–publication linker on COVID-NMA data

The 343 preprints tracked by COVID-NMA listed in doi-preprint-list.tsv were fed to the preprint–publication linker (preprintPublicationLinker.py) on October 23, 2020. The output was stored in doi-preprint-list.txt. These data were further analysed in the 20201104-evaluation-COVID-NMA.xlsx spreadsheet to compute evaluation results (Fig. 6).

## Appendix 3: Evaluation of the preprint–publication linker on Crossref data

The 4800 randomly selected preprint–publication links were downloaded from Crossref (cr-pplinks.py) on January 9, 2021. The 4693 preprints listed in doi-preprint-list.tsv were fed to the preprint–publication linker (preprintPublicationLinker.py) on January 10, 2021. The output was stored in 2017-2020_preprintPublicationLinker.txt. These data were further analysed in the 20210110_evaluation-Crossref.xlsx spreadsheet to compute evaluation results.

## Footnotes

1 https://www.who.int/emergencies/diseases/novel-coronavirus-2019.

2 See https://github.com/nicholasmfraser/covid19_preprints for regular updates.

3 https://covid-nma.com.

4 https://www.doi.org/registration_agencies.html.

5 https://www.cshl.edu/partner-with-us/preprints/.

6 https://api.biorxiv.org.

7 https://api.crossref.org.

8 The number of indexed records is provided at https://search.crossref.org.

9 https://iloveevidence.com.

10 https://covid-19.cochrane.org.

11 See https://www.crossref.org/education/metadata-stewardship/reports/doi-error-report/.

12 See the publication 10.1007/s11427-020-1732-2 and the erratum 10.1007/s11427-020-1751-3.
